# Farming, Q fever and public health: agricultural practices and beyond

**DOI:** 10.1186/s13690-017-0248-y

**Published:** 2018-01-06

**Authors:** Marcella Mori, Hendrik-Jan Roest

**Affiliations:** 1 Bacterial Zoonoses of Livestock, Veterinary and Agrochemical Research Centre, CODA-CERVA, Brussels, Belgium; 2 Department of Bacteriology and Epidemiology, Wageningen Bioveterinary Research, Lelystad, the Netherlands

**Keywords:** *Coxiella burnetii*, Agricultural practices, Transmission, Surveillance, Control, One health

## Abstract

Since the Neolithic period, humans have domesticated herbivores to have food readily at hand. The cohabitation with animals brought various advantages that drastically changed the human lifestyle but simultaneously led to the emergence of new epidemics. The majority of human pathogens known so far are zoonotic diseases and the development of both agricultural practices and human activities have provided new dynamics for transmission. This article provides a general overview of some factors that influence the epidemic potential of a zoonotic disease, Q fever. As an example of a disease where the interaction between the environment, animal (domestic or wildlife) and human populations determines the likelihood of the epidemic potential, the management of infection due to the Q fever agent, *Coxiella burnetii*, provides an interesting model for the application of the holistic One Health approach.

## Background

Humans had hunted herbivores for a long time, before they started to keep the slowest and relatively placid wild herbivores around their villages during the Neolithic period. Domestication allowed the transition from a hunter-gatherer human behavior to a sedentary lifestyle. Together with settlement, and an increased defense of the territory, the cohabitation with animals led to the emergence of epidemics associated with the building up of shared parasite/pathogen communities over the course of time [1]. Phylogenic studies show that domesticated animals were not just the source of pathological infections for humans but that they were also the recipients of pathogens that evolved from humans in the opposite direction. Examples are *Taenia* and *Mycobacterium bovis*, which originated from humans' consumption of raw carnivores/scavengers prey meat followed by a transfer to the domesticated animals and though an adaptation of a *Mycobacterium tuberculosis* strain to the animal recipient, respectively [1–3]. The majority of human pathogens known so far are zoonotic diseases, and their emerging/re-emerging property is associated with the feature of having a broad host range [4]. In general, ungulates seem to support the majority of human pathogens species and changes in agricultural practices is one of the major drivers associated with emergence and re-emergence [4]. The intensification of agricultural activities in terms of increased population size, density, low genetic diversity of races, and synchronization of hormonal cycles has indeed facilitated the role of livestock in the transmission of zoonotic diseases. Interaction with wildlife species attribute them also a role of intermediate and amplifier for emerging strains spreading from the sylvatic cycle. The evolution of farming practices from an extensive (genetically diverse) to an intensive (highly dense and with low genetic variability) management of livestock also provided new dynamics for disease transmission. Epidemiological models indicate that the probability of minor epidemics is associated with extensive farming while major epidemics or no epidemics at all are probable in the case of intensive husbandry [5]. Disease transmission dynamics evolve with human activities, including trade, but intensive farming has definitely contributed to the spread of pathogens that are transmitted through environmental pathways [6], especially via the airborne route. Whereas the implementation of biosecurity measures can mitigate between-herd disease spread of certain zoonotic diseases, these are not sufficient for aerosol transmitted pathogens, which through ventilator systems or natural ventilation and wind can spread into the environment and contaminate other herds, wildlife and ultimately humans. Epidemics of Q fever are, in part, an example of the interactions between agricultural practices and disease burden, and provide knowledge on risks and drivers of a zoonotic disease at the livestock-human interface. This article reviews some factors that influence the epidemic potential of Q fever such as those related to agricultural policies, environmental conditions, intrinsic features of the causal *Coxiella burnetii* bacterium and its relation with the cell host environment, as well as surveillance and control measures effected in a specific country. As an example of a disease where the interaction between animal (domestic or wildlife), environment and human populations determines the likelihood of the epidemic potential, the management of infection due to the Q fever agent, *C. burnetii*, provides an interesting model for the application of the holistic One Health approach.

## Q fever epidemics and farming

The pleomorphic etiological agent of Q fever, *C. burnetii* is an intracellular bacterium that replicates in the parasitophorous vacuole of the parasitized cell (generally a macrophage or a trophoblast). The parasitic activity of this bacterium consists of building a finely orchestrated machinery that diverts the normal metabolism of the parasitized cell to become a surviving container devoted to the proliferation of the bacterium [7]. *C. burnetii* has a broad host range (with a role in dissemination or maintenance of the disease) and is found in unicellular organisms, invertebrates, birds and mammals [8]. Domestic ruminants are the primary reservoir for human infection, and the majority of human epidemics are related to exposure to small ruminant (sheep and goats) infected products (placenta membranes, birth fluids, animal excretions or contaminated dust) [9, 10]. Transmission of infection from animals to humans is facilitated by the inhalation of contaminated aerosols. The infection in animals is usually sub-clinical or asymptomatic except in pregnant animals where it can cause abortion and stillbirth. Highly infected placentas can be retrieved from abortions but also from the natural parturition of infected animals [11]. The estimated bacterial load in infected placenta can be as high as 10^9^ bacteria/g [12]. As the estimated infective dose via the aerosol route is slightly above 1 bacterium [13] then exposing people to these highly infected animal products represents a high risk for infection. In addition, if several herds are affected simultaneously over a wide geographical area, the conditions for abortion storms can be created, which increases the likelihood for human epidemics [14].

The occurrence of human Q fever is mainly sporadic, most often limited to at risk individuals (abattoir workers, farmers, veterinarians), and rarely epidemic [9, 10]. Modifications in farming practices, which also imply expansion of trading, have been related to Q fever epidemics in humans, particularly when the activity was implemented in an area in close proximity to the human population.

In Bulgaria, a collapse of the state-owned and cooperative cattle/sheep farms in the 1990s opened the doors to the goat farming. This change in the agricultural sector was associated with an increased incidence of human Q fever cases [15]. Individual farmers started to raise goats as a form of readily available cheap food for private consumption. As a consequence the number of goats tripled in a few years, creating the conditions for a potential epidemic context. While cattle and sheep were kept separate from the human population, goats were reared in close proximity to farmers and their families. The goats passed daily through villages and small towns to go to the pastures, hence spreading contaminated aerosol around and infecting unrelated bystanders [16].

In the Netherlands, goat farming started to increase after the introduction of the European milk quota system for dairy cattle, and following two consecutive outbreaks of classical swine fever and foot-and-mouth disease that affected the pig and the cattle industries respectively [17]. There was about a 50-fold increase in the number of goats between 1983 and 2009 [17], leading to a massive importation of animals, possibly from contaminated sources. The rearing of goats was mostly indoors and intensive, with farms having several thousands of animals. In these contexts, infection with endemic and/or imported *C. burnetii* lead to abortion waves and the excretion of high numbers of bacteria in the environment, and ultimately dissemination to the general population.

The notion that geographical spread of Q fever worldwide was linked to the traffic of infected animals has also been shown. In Slovakia, the first epidemics directly linked to imported infected sheep have been described since 1954 [15]. Uncontrolled movements of animals for the wool industry or for livestock production have contributed to the establishment of domestic coxiellosis in this country [15, 18]. More recent studies have tried to model and quantify the influence of trade on the introduction of new Q fever infection within a herd. Animal movements is a certain risk factor and the higher the number of herds from which a herd receive animals, the higher the risk of infection [19]. Within herd and between-herd Q fever prevalence are variables that influence the magnitude of this risk [20]. If within herd prevalence is high, the purchase of a few animals from this infected herd will result in a high probability of the infection being introduced to the purchaser's herd. If the effect is possibly negligible in case of high between-herd prevalence, then there will instead be a large probability of disease if the between herd prevalence is small [20]. In other words, if the Q fever prevalence in a defined country is low, the impact of buying even just a few animals from infected sources will be important and could be the likely source of large outbreaks [21]. In a high local animal density area (for example for cows of about 60–100 animals per km^2^) with a high Q fever prevalence, the trade seems to explain only a low proportion of the total incidence, while a major contribution to infection is linked to other transmission routes such as airborne transmission [21].

### Environment and geographical landscape

There are various transmission routes of *C. burnetii* to humans. These include well established and effectives routes, such as inhalation of aerosolized bacteria spread from infected reservoirs [9], and other seldom recorded routes including food consumption (unpasteurized milk and derivatives [22]), tick bite [23], and human-to-human [24, 25]. *C. burnetii* can persist for long in the environment, resist to physical and chemical stresses, and easily dispersed due to a pseudo-sporulation process [26]. Outbreaks or cluster cases related with aerosolized transmission route are frequent and often associated with specific environmental conditions favoring the diffusion [27–32], including windy days [27–29, 32, 33], landscape [33] or artificial circumstances [30]. In France, the mistral, a strong west-northwest/north wind that might blow for consecutive days, also on sunny and dry days, was an underlying cause of the dispersal of sheep *C. burnetii* contaminated products and the insurgence of Q fever human cases in the southern sheep grazing area [27]. Artificial means of dispersion, for instance mediated by air movement from helicopters, was related to a Q fever epidemic originating from neglected accumulation of sheep waste from a nearby abattoir [30]. Low speed wind in the flat area of North Brabant was a good meteorological condition for *C. burnetii* dispersion during the outbreaks in the Netherlands [33]. Climate, vegetation, land use, and soil humidity are intersected geographic local factors that influence transmission. Vegetation that provides shelter for dust particles and limits the speed of the wind exerted an influence during the Dutch outbreak [34]. Infected particles from farms with a surrounding low vegetation density had a higher probability of being dispersed and transmitting Q fever to the local population (within the 5 km buffer zone). Hence, farms with wetter average soil moisture conditions were likely to provide less risk for human Q fever infection within the direct vicinity of the infected source. This relation was probably due to the influence of wet soils on dust production and deposition. Wetter soils are less sensitive to wind erosion and have higher vegetation densities and therefore provide a certain level of protection against airborne transmitted particles [34].

Apart from wind contribution, close proximity to the infective source has been described as one main risk factor in various epidemics of Q fever, including that in the Netherlands [35–38]. In the context of a focal point source, the risk resides within a few dozen meters [35]; in case of regional Q fever abortion waves between farms, the 4–5 km zone from the highly densely infected farm is associated with the highest attack rate in the human population [37].

### Bacterial evolution and virulence

As it is an intracellular bacterium with large spectrum of hosts, *C. burnetii* has continuously remodeled its genetic structure during its evolution. Hypothetically, this recognized species within the *Coxiella* genus has originated from a soft-tick symbiont ancestor, following a process implying significant genetic exchanges, horizontal gene transfer, and genome acquisitions [39]. The process of how the *Coxiella-like* tick endosymbionts become the vertebrate pathogen *C. burnetii* has not yet been fully elucidated. One hypothesis is that most of the vertebrate tropisms factors were acquired directly from eukaryotic cells while other pathogenic islands were acquired via lateral gene transfer from co-infecting tick pathogens [39]. Expansion of insertion sequences elements, accumulation of pseudogenes and other genetic rearrangements have added to the generation of strains with different virulence [40]. The major virulent factor recognized for *C. burnetii* is its full-length lipololysaccharide (LPS); the bacterium is then present in its phase I antigenic form. Artificially derived variants of *C. burnetii* with truncated LPS (phase II form) retain the lipid A and the sugar core but no *O*-antigen and are avirulent in a rodent infection model [41–43]. Besides this antigenic shift, resembling to the transition smooth-rough of other intracellular bacteria, genetic analyses have demonstrated a relationship between genome content and pathogenic variants of *C. burnetii* [44]. Some pathotypes for instance are more associated with a chronic course of the disease, while others are never found or have rarely been isolated in humans [44]. The magnitude of an unprecedented epidemic of Q fever, such as that which occurred in the Netherlands, has led to the circulation of a hyper-virulent pathotype being proposed [45]. How these phenotypic features relate to specific genetic traits are increasingly being determined thanks to the axenic growth of the bacterium [46] and the availability of full genome sequences of strains. Following internalization of *C. burnetii* in the host cell, the bacterium manipulates several host-cell processes to subvert the normal cellular physiology and produce the parasitophorous vacuole needed for the bacterial proliferation. The Dot/Icm T4BSS secretion system releases proteins with effector functions that reorganize the cell compartments to obtain productive infection [47, 48]. There are now twenty-four mutants in effector proteins known to provoke a defect in intracellular growth and/or vacuole formation [7]. In addition, genome-wide screening with Himar-1 Tn transposon identified additional hundreds of mutants in genes encoding Dot/Icm structural proteins, chaperones, regulators, and effectors that have an impact on intracellular growth [7].

### Host-pathogen interactions

*C. burnetii* displays a wide range of cell type tropism including but not limited to monocyte and macrophages, trophoblasts, adipocytes and epithelial cells [10, 49, 50]. The bacterium internalizes by binding to the αvβ3 integrin of the targeted cell, and in monocyte/macrophages stimulates their activation through binding to Toll-like receptors (TLR) [51]. In humans, polymorphism in TLRs (TLR1, NOD2, TLR6 and TLR10) might be associated with the reduced production of cytokines after infection and finally less responsiveness of macrophages to infection [52]. It is now quite clear that the adaptive immune response plays the major role in controlling *C. burnetii*, albeit that the two arms (cell-mediated and antibodies) do not seem to provide an equal contribution: while Th1-type is essential to control the infection, antibody production can be dispensable. The innate pro-inflammatory response plays a role in granuloma formation and neutrophils are required for both the host immune response to primary infection as well as the vaccine-induced protection against *C. burnetii* as shown in a neutrophil-depleted mouse model of infection [53]. The cytokine pattern of pro-inflammatory cytokines and finally immune response as a whole can be differently stimulated depending on the isolate-type [54]. Human PBMCs produce significantly higher amounts of IL-1β, TNF-α, and IL-22 upon stimulation with bovine strains than after goat and sheep strain stimuli. While IFNγ, which has an essential microbicidal activity against *C. burnetii*, was similarly induced by all strains; the difference in production for other cytokines might indicate a different activation of other essential immune pathways necessary for *C. burnetii* control [54]. On the other hand, upregulation of IL10 and prostaglandin E_2_ was correlated with persistence of *C. burnetii* infection [55]. Virulence factors of the bacterium and the immune host competence therefore define the susceptibility to infection and likely outcome of epidemics.

### Surveillance systems for Q fever in animals

**Fig. 1:**
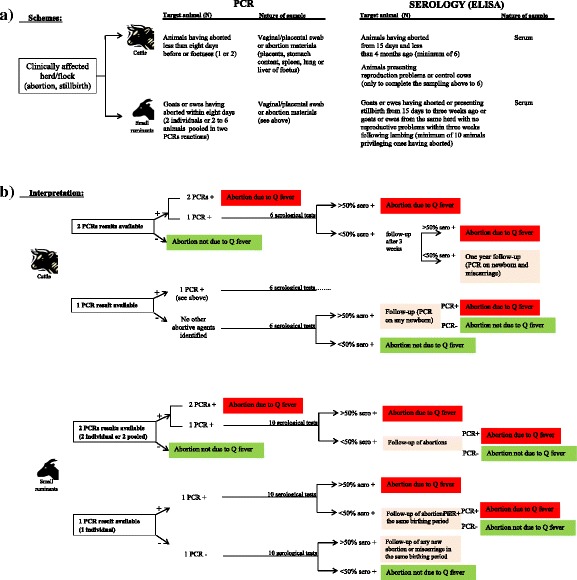
Passive surveillance schemes (a) and results’ interpretation (b) for Q fever in animals (as suggested from [56])

Monitoring of Q fever in animals is important for providing optimal animal health conditions, for retrieving the information needed for assessing the implementation and effectiveness of control programs, for providing a platform for early case detection, and ultimately for ensuring readiness in the event of an epidemic. Harmonized schemes to be applied in endemic regions or countries have been proposed for ruminants, which include passive and active surveillance, whether in cattle or small ruminants [56] (passive surveillance summarized in Fig. 1).

In the case of cattle passive surveillance, clinical signs need to be taken into account, particularly when series of abortions occur. In this case, vaginal swabs of one or two animals having aborted less than eight days before or abortion material can be tested by direct (PCR) diagnosis. Furthermore, serological blood sampling of at least six animals having aborted more than 15 days previously or presenting reproductive problems within the herd should be considered. Passive surveillance in small ruminants follows a similar approach to cattle. Clinical signs indicating abortions at the end of gestation and/or premature birth as well as the presence of necrotic placenta [11] should lead to Q fever being included in the differential diagnosis of the abortive agent. Following a series of abortions, vaginal swabs of two to six animals having aborted less than eight days before or abortion material can be tested with PCR. If there is a lack of material for PCR screening, serological samples of at least ten animals of the affected herd should be considered, which includes clinically infected animals or those having given normal birth [56]. Interpretations of results from these schemes are summarized in Fig. 1 [56].

Active monitoring schemes are cost-intensive but they are the appropriate choice if disease prevalence in animals and/or in humans is high. Different sampling strategies can be used and these should reflect the epidemiological situation of a specific country [56]. For Belgium, a mandatory notification of abortion (cattle and small ruminants) applies and a set of abortive agents is included in the differential diagnosis. For cattle, Q fever detection by PCR was included in the set from 2010 to 2015. In small ruminants, screening of abortion for the Q fever agent has been in force since 2010, as a consequence of the Dutch Q fever outbreak. When abortion material is not available for PCR testing, serological diagnosis of animals that have aborted is applied for diagnosis. In addition to abortion screening, regular bulk tank milk (BTM) monitoring is in place for all dairy small ruminant farms, comprising both serological (ELISA) and PCR testing. In the Netherlands, the active surveillance system is restricted to dairy sheep and goat farms and includes monthly BTM monitoring by PCR detection of DNA of *C. burnetii* [17].

Positive PCR results of abortion and/or of BTM screening are notified within the 48 h to the competent national body, namely the Federal Agency for the Safety of the Food Chain (FASFC) [57] for Belgium and the Netherlands Food and Consumer Safety Authority (NVWA) in the Netherlands [17]. Compulsory control measures are then undertaken in the infected small ruminant herd including the vaccination of the herd and restricting the selling of raw milk and the derived dairy products [58].

## Options to control Q fever

Q fever can lead to clinical disease in humans and animals. As domestic ruminants are most frequently the source of Q fever in humans, the control of Q fever in the animal reservoir will also lead to a reduction of Q fever in the human population as the exposure will be reduced. However, as Q fever can have an economic impact on the farm level, the presence of Q fever symptoms in small ruminant or cattle herds might also be an incentive to control *C. burnetii* infection in these herds [59].

Control options can be divided into four main groups: 1) measures to identify infected farms; 2) measures to reduce excretion of *C. burnetii*; 3) measures to reduce the dispersion of *C. burnetii* and 4) measures to reduce human exposure [14, 17].

Measures to identify infected farms, such as the obligation to notify symptoms of Q fever, abortion, or a positive BTM result to the government, are especially needed to control Q fever in humans. A proper overview of the farms is needed to identify exposure sources of human Q fever  and to facilitate epidemiological evaluation and risk assessment. In addition, when control measures become part of legislation, there is a need to supervise the implementation and follow up of the measures. In most countries, Q fever is a notifiable disease in humans as well as in animals [14]. A prerequisite for this is the availability of adequate diagnostic tests and awareness of the presence of the disease by general practitioners and veterinarians. Notification criteria may vary per country, but notification of abortions is always one of the criteria.

Measures to reduce the excretion of *C. burnetii* are important for the control of human Q fever as well as controlling Q fever on the farm level. Vaccination with a phase 1 vaccine is effective in reducing abortions as well as the excretion of *C. burnetii* [60, 61]. Another approach in reducing the excretion of *C. burnetii* is to prevent possible excretion of *C. burnetii* by infected pregnant ruminants. This can be done by either preventing them from becoming pregnant via a breeding ban or preventing them from giving birth by culling infected pregnant ruminants. Both measures were implemented during the Dutch Q fever outbreak as an ultimate goal in preventing the excretion of *C. burnetii* from goat farms [17]. Modeling studies on the effectiveness of control measures suggest that vaccination is the most effective long-term intervention to prevent excretion of *C. burnetii* on goat farms [62]. Vaccination is also effective in preventing shedding of *C. burnetii* in infected dairy cattle herds [59].

Measures to prevent the dispersion of *C. burnetii* focus on stopping the spread of the bacteria over a larger area. When infected goats are transported to other herds, *C. burnetii* will travel with them. Transport of manure from infected herds might also facilitate dispersion over a wider area. A transport ban of infected goats and manure from infected farms will prevent the spread via these routes. In several countries, measures to reduce the risk of manure in the spread of *C. burnetii* have been implemented varying from rules on how to handle manure to disinfection. The role of manure in the transmission of *C. burnetii* to humans, however, is under debate and conflicting studies have been published [63, 64]. Additional hygienic measures like the removal of risk material (placentas, aborted fetuses), indoor parturition and disinfection of parturition areas will also prevent the dispersion of *C. burnetii*.

Measures to reduce human exposure include the three main groups mentioned above. Additionally direct transmission from infected animals to humans can be prevented by avoiding direct contact between humans and infected animals. In the Dutch outbreak, a visitor ban on Q fever positive dairy goat farms was implemented. As Q fever is considered to be an occupational disease, this measure might be quite important in preventing employees in the animal husbandry sector from becoming infected with Q fever. However, the limitations of preventing transmission are shown in a study among culling workers in the Netherlands: despite the use of personal protective equipment culling workers still became infected, as 17.5% seroconverted for antibodies to *C. burnetii* [65].

The above-mentioned measures are, in variable arrangements, part of the control of Q fever in several countries. However, the effectiveness of the individual measures remains unclear, as the number of studies assessing the effectiveness is limited. The studies published so far reveal that vaccination is effective in reducing abortion and the excretion of *C. burnetii* [60, 61]. Studies on the effectiveness of control arrangements are rather descriptive; the measures taken in the Dutch Q fever outbreak seem to be most broad and far-reaching and resulted in a reduction of human Q fever [17].

### One health approach in Q fever control

The One Health approach can be defined as ‘the collaborative efforts of multiple disciplines to optimize human, animal, and environmental health’. A crucial aspect of this approach is that the different disciplines working on human, animal, and environmental health collaborate and integrate their knowledge, while taking into account the nature of the three domains. The goal is to realize better solutions for the system as a whole compared to a monocentric approach. In the control of zoonotic diseases in humans, the One Health approach can be really well applied, as it takes into account knowledge from the human, veterinary, and environmental domains. Q fever is an example of this ‘par excellence’: Humans become infected from the animal reservoir from which the Q fever bacterium is transported to humans via the environment [66].

Prior to the large Dutch Q fever outbreak, Q fever local cases in the Netherlands were limited to a few cases per year [67]. Q fever was also known to be present in the animal populations but did not cause significant disease. This changed in 2005, when the first abortions in dairy goats were registered and in 2007, when the first human Q fever outbreak in the Netherlands was recorded. Between 2005 and 2009 abortions on 28 dairy goat farms and 2 dairy sheep farms were detected and between 2007 and 2010 about 4000 human cases were notified [17]. Collaborative action from the human and animal domains was an important factor in controlling the disease. Joint expert meetings organized during which effective control measures for public health were discussed. Research efforts were also combined to identify the cause of the human disease, for example. Genotyping of *C. burnetii* in clinical samples from goats and humans revealed that one strain was clonally spread from goats to humans [45, 68].

The collaborative action, however, was not evident from the start of the outbreak. As Q fever abortions have only been notifiable since 2008, the exchange of information about abortion and farm data before that date were restricted by privacy legislation. This frustrated the exchange of information between the animal and human domain and hampered the identification of sources of human Q fever cases. It also became clear that the mode of action and the consequences of measures were different in the public health and animal domains. Public health control is focused on advising both public bodies and private individuals, whereas in the animal sector control measures are imposed on farms i.e. on enterprises. These enterprises have a legal status and can litigate the government for the imposed control measures. Therefore these measures have to be risk and science based. This difference in approach between the public health and animal health domains lead to misunderstandings in the willingness to control the disease. However, during the course of the ongoing epidemic this risk-based approach failed to show the desired result. A change in approach towards the precautionary principle was needed to implement drastic measures like the culling of pregnant goats on infected farms so that the Dutch Q fever epidemic could finally be overcome [69].

One of the lessons learned from the Dutch Q fever outbreak is that collaborative action is needed to combat a complex disease [70]. That was also the conclusion of the official evaluation of the Q fever outbreak in the Netherlands [71]. This has resulted in the establishment of a national zoonosis structure with a signaling forum that meets monthly. This signaling forum is comparable to the Human Animal Infections and Risk Surveillance group (HAIRS) in the UK [72]. Its goal is to assess the risk of disease signals from both the human and the veterinary field at an early stage so that action can be taken to avoid the emergence of outbreaks.

## Conclusion

Q fever is a disease of animals and humans. *C. burnetii* has large host spectrum and its transmission dynamics, survival, and maintenance in the environment make the biology of this pathogen quite complex. Control and management of the disease definitely requires a holistic approach. That can be achieved by gathering expertise from multiple scientific disciplines and stakeholders so that the expectations farmers and patients but also the wider community can be met.
